# Basis for the Induction of Tissue-Level Phase-2 Reentry as a Repolarization Disorder in the Brugada Syndrome

**DOI:** 10.1155/2015/197586

**Published:** 2015-10-25

**Authors:** Alfonso Bueno-Orovio, Elizabeth M. Cherry, Steven J. Evans, Flavio H. Fenton

**Affiliations:** ^1^Department of Computer Science, University of Oxford, Oxford OX1 3QD, UK; ^2^School of Mathematical Sciences, Rochester Institute of Technology, Rochester, NY 14623-5603, USA; ^3^The Heart Institute, Beth Israel Medical Center, New York, NY 10003, USA; ^4^School of Physics, Georgia Institute of Technology, Atlanta, GA 30332-0430, USA; ^5^School of Biology, Georgia Institute of Technology, Atlanta, GA 30332-2230, USA; ^6^School of Applied Physiology, Georgia Institute of Technology, Atlanta, GA 30332-0356, USA

## Abstract

*Aims*. Human action potentials in the Brugada syndrome have been characterized by delayed or even complete loss of dome formation, especially in the right ventricular epicardial layers. Such a repolarization pattern is believed to trigger phase-2 reentry (P2R); however, little is known about the conditions necessary for its initiation. This study aims to determine the specific mechanisms that facilitate P2R induction in Brugada-affected cardiac tissue in humans. *Methods*. Ionic models for Brugada syndrome in human epicardial cells were developed and used to study the induction of P2R in cables, sheets, and a three-dimensional model of the right ventricular free wall. *Results*. In one-dimensional cables, P2R can be induced by adjoining lost-dome and delayed-dome regions, as mediated by tissue excitability and transmembrane voltage profiles, and reduced coupling facilitates its induction. In two and three dimensions, sustained reentry can arise when three regions (delayed-dome, lost-dome, and normal epicardium) are present. *Conclusions*. Not only does P2R induction by Brugada syndrome require regions of action potential with delayed-dome and lost-dome, but in order to generate a sustained reentry from a triggered waveback multiple factors are necessary, including heterogeneity in action potential distribution, tissue coupling, direction of stimulation, the shape of the late plateau, the duration of lost-dome action potentials, and recovery of tissue excitability, which is predominantly modulated by tissue coupling.

## 1. Introduction

The Brugada syndrome (BrS), first described as a new clinical entity in 1992 [1], constitutes a distinct subtype of idiopathic ventricular fibrillation with unique electrocardiographic (ECG) manifestations characterized by right bundle branch block, ST-segment elevation of coved or saddle-back type, and T-wave inversion in the right precordial ECG leads *V*_1_ to *V*_3_, together with a high incidence of sudden death from ventricular tachyarrhythmia [1–3]. It is believed that it causes 4–12% of all sudden cardiac deaths and up to 20% among patients without identifiable structural abnormalities [4]. A landmark in the characterization of the disease was the identification in 1998 by Chen and coworkers [5] of the first mutation in BrS patients in the SCN5A gene, which encodes the *α*-subunit of the cardiac sodium channel. Since then, a total of 14 BrS subtypes have been detected according to their genetic basis, linked to either a loss of function in sodium or L-type calcium currents or increased capacity of transient outward, delayed rectifier, or ATP-sensitive potassium channels [6]. Despite substantial progress in the identification and characterization of the syndrome over the past two decades, implantation of a cardioverter defibrillator is still the only established effective treatment for the disease [7].

Two main mechanisms have been postulated to explain the ECG features and the arrhythmogenic nature of BrS: the repolarization disorder and the depolarization disorder hypotheses [8]. The* repolarization disorder* hypothesis focuses on the unequal expression of the transient outward current (*I*_to_) in the right ventricular epicardium, leading to a heterogeneous reduction of the epicardial action potential (AP) dome, marked dispersion of repolarization, and vulnerability to arrhythmia induction in the form of phase-2 reentry (P2R) [9–11]. On the other hand, reductions of sodium current availability or other factors affecting conduction reserve (such as the presence of abundant adipose tissue or fibrosis) are believed in the* depolarization disorder* hypothesis to amplify conduction delays in the right ventricular outflow tract, constituting the substrate for arrhythmia [8, 12, 13]. In close connection with the depolarization disorder hypothesis, discontinuous conduction of the AP has also been suggested as a possible trigger of P2R in BrS [14, 15]. The repolarization hypothesis has experimental confirmation in canine right ventricular wedge preparations [11, 16], in the recording of more prolonged activation-recovery intervals in epicardial tissue than in the endocardium of BrS patients after administration of pilsicainide [17], and in repolarization heterogeneity maps in BrS patients [18]. On the other hand, recent* ex vivo* [19] and* in vivo* [20] studies have revealed significant conduction delays in the right ventricular outflow tract of BrS subjects, but not epicardial reduction of the AP dome, giving support to the depolarization hypothesis.

Although not neglecting the likely contribution of depolarization and conduction abnormalities in explaining the manifestations of the disease, the aim of the present study is to use mathematical modeling and computer simulation to determine the conditions necessary for P2R reentry to occur at the tissue level in the context of the BrS as a repolarization disorder. Testing of our hypotheses requires a human ventricular AP model that is in agreement with experimental recordings and that allows the independent alteration of AP morphology by varying specific parameters. This is made possible in our study by using an extension of our previous human ventricular myocyte model [21]. Despite not incorporating a detailed description of the main sarcolemmal ionic currents that may be affected by the disease, the model provides an accurate representation of the human ventricular AP in terms of its morphology and rate-dependent properties [21]. In this regard, our modeling approach can be considered independent of a specific genetic basis of BrS, since different mutations may result in similar alterations of the AP at the tissue level [6, 7]. Reentry patterns induced by BrS are simulated in one-dimensional cables, two-dimensional tissue layers, and reconstructed three-dimensional ventricular geometries. The contributions of multiple factors in initiating P2R, including AP morphology, tissue coupling, and recovery of excitability, are analyzed.

## 2. Materials and Methods

### 2.1. Mathematical Model

The computational model used in the present work is based on the human ventricular myocyte model of [21]. The model accounts for the sum of all transmembrane currents into three main categories (*J*_fi_, fast inward current; *J*_si_, slow inward current; and *J*_so_, slow-outward currents), representing an integrated flux of sodium, calcium, and potassium currents into the ventricular myocyte, respectively. To facilitate reproduction of the prolonged AP domes associated with BrS, several minor modifications were made. Specifically, five independent threshold parameters (*θ*_*v∞*_, *θ*_*w∞*_, *θ*_so_, *θ*_si_, and *θ*_*s*_) were used in the Heaviside functions for *v*_*∞*_, *w*_*∞*_, *J*_so_, *J*_si_, and *τ*_*s*_ (resp.), rather than requiring these threshold parameters to be the same as parameters used in other equations (*θ*_*v*_^−^, *θ*_0_, *θ*_*w*_, *θ*_*w*_, and *θ*_*w*_, resp.). In addition, the following time constants were reformulated to be dependent on their associated gating variables:

(1) τw+=τw1++τw2+−τw1+1+tanh⁡kw+w−wc+2,τsi=τsi1+τsi2−τsi11+tanh⁡ksis−sc2.

The modified equations can be used in conjunction with the previous model by setting *θ*_*v∞*_ = *θ*_*v*_^−^, *θ*_*w∞*_ = *θ*_0_, *θ*_so_ = *θ*_si_ = *θ*_*s*_ = *θ*_*w*_, *τ*_*w*2_^+^ = *τ*_*w*1_^+^, and *τ*_si2_ = *τ*_si1_. In the equations, the voltage variable *u* varies between 0 and 1.4 and is rescaled to its physiological range in mV as *V*_mV_ = 85.7*u*–84 for comparison with experimental data [21].

Model parameter values were selected to replicate the observed morphological AP characteristics associated with BrS, including decreased phase 0 amplitude [22], slower upstroke [22], and prolonged dome [23]. A second model was also developed to reproduce the* in vivo* human BrS epicardial monophasic APs observed by Kurita et al. [24].  Table 1 lists parameter values for the two models of Brugada-affected epicardial cells used throughout the paper, except where noted otherwise. To produce lost-dome regions with significantly shorter APs, the initial component of the slow-outward (potassium-like) current was increased by reducing *τ*_so1_ 30% in Model 1 and 50% in Model 2. Parameter values for normal human epicardium, endocardium and midmyocardium were taken from [21].

### 2.2. Computational Methods

In one-, two-, and three-dimensional simulations, the cable equation:

(2) $$\begin{array}{c} \partial_{t}V=\nabla \cdot \left(\;D\nabla V\;\right)-I_{ion}+I_{stim} \end{array}$$

was integrated in time using an explicit Euler scheme with a time step of Δ*t* = 0.02 ms and a spatial resolution of Δ*x* = 150 *μ*m. Spatial derivatives were approximated using standard second-order finite differences and the diffusion coefficient was set to 1.171 cm^2^/s for human ventricular tissue [21]. No-flux boundary conditions were used. In the realistic right ventricular wedge geometry, no-flux boundary conditions were implemented using the phase-field method [25, 26].

Reconstructed transmural pseudo-ECGs were obtained by assuming an infinite volume conductor and calculating the dipole source density of the membrane potential *V* at all node points of the medium using the following equation [27]:

(3) $$\begin{array}{c} \text{ECG}=\int \frac{D\nabla V\cdot \vec{r}}{{\left\|\vec{r}\right\|}^{3}}dx, \end{array}$$

where $\vec{r}$ is the vector from the recording electrode to a point in the tissue. A 0.6 cm cable, comparable to the thickness of the right human ventricle, with a distribution of epicardial, endocardial, and midmyocardial cells similar to that of [28], was used in these calculations. The recording electrode was placed 0.25 cm from the epicardial end of the cable.

## 3. Results

### 3.1. Brugada Syndrome Action Potential Morphologies and Electrocardiograms

**Figure 1:**
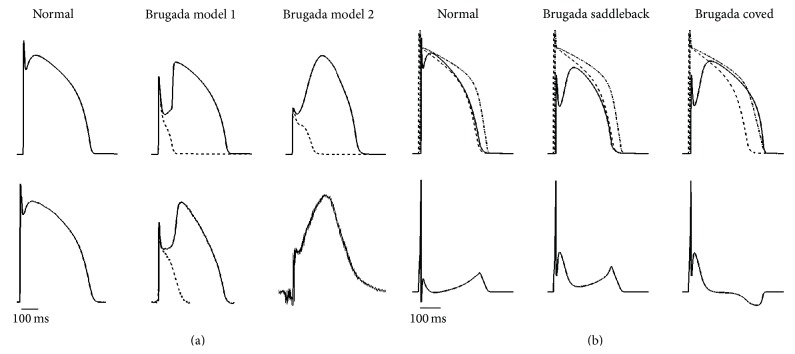
Validation of the mathematical model of Brugada syndrome (BrS). (a) Ventricular epicardial action potentials (APs). Top row shows simulated normal and BrS types 1 and 2 human epicardial models, with delayed dome (solid) and lost dome (dashed) in the latter. Bottom row shows experimental AP recordings (healthy human epicardial AP, adapted from [33], microelectrode recordings in a male dog using 5 *μ*M terfenadine, data courtesy of J. Fish and C. Antzelevitch, and* in vivo* human BrS epicardial monophasic APs, adapted from [24], resp.). (b) Reconstructed transmural pseudo-ECGs under normal and BrS conditions, together with epicardial (solid), endocardial (dashed), and midmyocardial (dash-dotted) APs. To obtain the two different morphologies of ST-segment elevation, Model 1 was used with *k*_si_ = 6 for the saddleback type and with *k*_si_ = 6 and *τ*_*w*2_^+^ = 180 for the coved type.

Through the modifications to the human epicardial cell model described above, we are able to reproduce Brugada-affected APs.  Figure 1(a) shows the normal epicardial AP and the two different BrS AP morphologies produced by Model 1 and Model 2. Both cases are characterized by a stunted upstroke, after which the prominent epicardial notch produces a strong repolarization. If the AP notch is especially strong or the AP upstroke is especially weak, the cell can be repolarized beyond the threshold for activation of the AP dome and produce a very short AP (dashed). In other cases (solid), the cell repolarizes to a more modest voltage, and the development of the AP dome during phase 2 is delayed, leading to a prolonged AP. Our models reproduce two types of Brugada-affected AP morphologies. Model 1 exhibits a long delay in the development of the AP plateau, after which a fairly rapid second depolarization phase occurs, followed by a later, more pronounced dome [29]. This type of AP morphology is similar to experimentally observed canine APs in the presence of terfenadine (Figure 1(a), central panel of bottom row). A second Brugada-affected morphology is replicated by BrS Model 2. In this case, the delay in the development of the plateau is not nearly so pronounced. This type of morphology, which has been observed in monophasic AP recordings from the right ventricular outflow tract in humans [24], may be more likely when the sodium current is especially small but *I*_to_ is not large enough to delay activation of calcium channels.

The BrS AP models, used in combination with normal endocardial and midmyocardial cells [21], are able to reproduce ECG characteristics associated with the syndrome (Figure 1(b)). Compared to the normal ECG, Brugada-affected ECGs are characterized by ST-segment (J-point) elevation [30] and can exist in two different morphologies: saddleback and coved. The coved-type morphology is required for diagnosis, while the saddleback-type morphology requires confirmation using pharmacological challenge (conversion into coved-type) or genetic analysis. To account for the negative T wave in coved-type ST elevation, prolongation of the epicardial AP dome is needed [24, 30]. We are able to reproduce both types of morphologies.

### 3.2. Mechanism of Phase-2 Reentry

On its own, the delay in dome formation leads to AP prolongation and creates a dispersion of repolarization and refractoriness. In combination with regions where the AP dome is lost entirely, an ideal substrate for the development of P2R is provided. The diffusive effects produced by the large voltage gradients between lost-dome and delayed-dome sites can produce a very closely coupled extrasystole via P2R, which arises from phase 2 of the AP where the dome is maintained and propagates into the region where the dome is lost [11, 16, 22, 31, 32]. In this way, the ionic mechanism underlying BrS as a repolarization disorder not only provides the substrate for reentry in the form of epicardial and transmural dispersion of repolarization but also provides its own extrasystole to trigger the arrhythmia.

**Figure 2:**
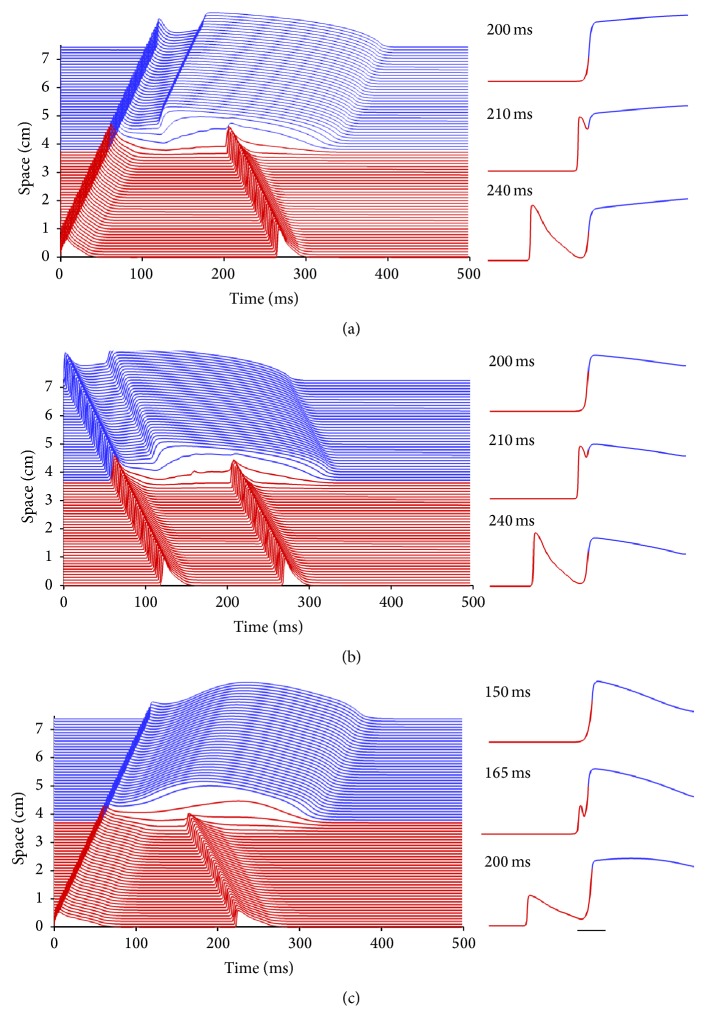
Space-time plots and wave profiles for three cases of phase-2 reentry (P2R) in one-dimensional epicardial cables. (a) Antidromic (retrograde) P2R generated by a propagating wave initiated from the lost-dome region (red) using Model 1. (b) Orthodromic (forward) P2R generated by a propagating wave initiated from the delayed-dome region (blue) using Model 1. (c) Antidromic P2R generated by a propagating wave initiated from the lost-dome region (red) using Model 2. In all cases, P2R originates slightly away from the interface between the lost-dome and delayed-dome regions (horizontal scale bar, 1 cm).

Using the formulations for prolonged and abbreviated APs, P2R can be induced in our BrS models in a one-dimensional cable (see Figure 2) containing a region where the dome is lost adjoining a region where the dome is delayed. In Figure 2(a), the cable is stimulated from the lost-dome region. Because the APs in this region are particularly short, large voltage gradients arise in the interface between lost-dome and delayed-dome regions. If the lost-dome region recovers from its AP while the delayed-dome region remains depolarized, diffusive currents can bring the recovered delayed-dome region above threshold and initiate a second propagating pulse, which is the P2R. In this case the P2R is antidromic, with propagation in the lost-dome region occurring in the opposite direction of the initial activation. P2R also can be orthodromic, as shown in Figure 2(b). In this case, the cable is stimulated from the delayed-dome region. The APs in the lost-dome region are short enough that the delayed-dome region is still sufficiently depolarized to initiate a second pulse after the lost-dome region has recovered. Model 2 is able to produce P2R in the same manner, as shown in the antidromic example in Figure 2(c).

### 3.3. Factors Affecting Inducibility of Phase-2 Reentry

**Figure 3:**
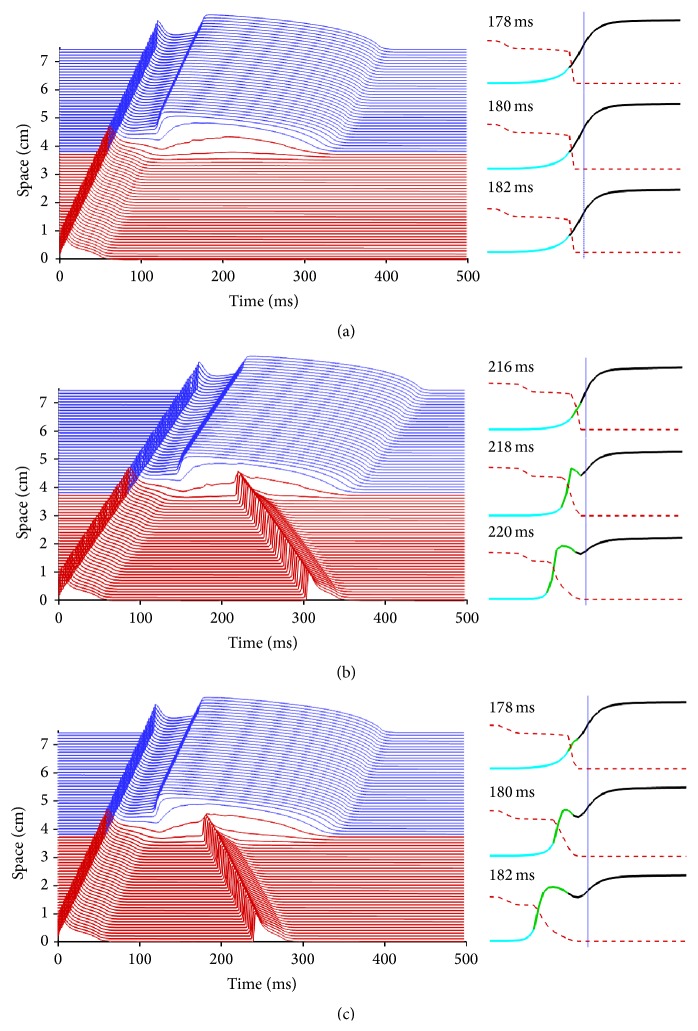
Dependence of the induction of phase-2 reentry (P2R) on different factors. (a) Unsuccessful attempt to initiate P2R. In this case, the action potential was prolonged, and sodium availability in the lost-dome region (red) has not recovered sufficiently for P2R to occur. (b) Successful P2R facilitated by reduced electrical coupling: other parameters remain as in (a). (c) Successful P2R under the same conditions as (a) except for a slight increase in the delay before formation of the dome in the delayed-dome region (blue). Wave profiles show details in the 1 cm region around the lost-dome and delayed-dome interface. Black indicates excited areas, light blue areas with fast inward channel availability, and green areas where fast inward channels are available and voltage has been brought above activation threshold. The red dashed line shows the fast inward current inactivation gate. For P2R to occur, part of the lost-dome region must repolarize, recover from fast inward channel inactivation, and then be brought above the excitation threshold through diffusive currents generated by the delayed-dome region.

The development of P2R requires a balance of multiple factors as we describe below. Otherwise, P2R cannot be induced even when pronounced changes in AP by the BrS are present. For example, Figure 3(a) shows a case where APs in the lost-dome region are slightly longer than those in Figure 2(a) (*τ*_so1_ is reduced only by 20%). In this case, the APs in the lost-dome region have not recovered sufficiently for the delayed-dome sites to initiate a propagating pulse, although locations near the delayed-dome region still experience significant depolarization from diffusive currents. However, P2R can be recovered even under these circumstances by decreasing cellular coupling.  Figure 3(b) demonstrates successful generation of P2R using the same parameter values as in Figure 3(a) but with the diffusion coefficient decreased by 40%. As a result, propagation slows, and there is more time for the lost-dome region to recover while the delayed-dome region is still capable of producing diffusive currents that bring the lost-dome region above the threshold of excitation, resulting in P2R.

It is also possible to recover P2R by other means involving the voltage profile of Brugada-affected APs. In Figure 3(c), P2R occurs using the same parameters as Figure 3(a) but with an increase in the time constant of the slow inward gate (−10%  *τ*_*s*1_). This change affects the AP dome in the delayed-dome region by further delaying the formation of the dome slightly, with little effect on the lost-dome region. The additional delay provides enough time for the lost-dome region to recover excitability, so that P2R occurs.

In all these cases, the primary mechanism by which P2R arises can be explained in a straightforward way, independent of the model description used. For P2R to arise, three events must occur in succession. First, part of the lost-dome region close to the interface between the lost-dome and delayed-dome regions must repolarize fully (or nearly so). Second, the inactivation gate of the fast inward current must recover above its recovery threshold, a process driven by membrane potential repolarization. Third, the delayed-dome region must produce enough diffusive current to reach this area in the lost-dome region and elevate again the membrane potential above the excitation threshold. Then, because the fast inward current is no longer inactivated, a full AP can be produced and can propagate unidirectionally to neighboring tissue. The right panels of Figure 3 show the generation of P2R in these terms, by presenting expanded views of the interface between the lost-dome and delayed-dome regions (1 cm scale bar in Figure 2). In these panels, black indicates excited regions during the initial AP, light blue indicates areas that have repolarized fully with recovery from fast inward current inactivation, and green indicates areas where diffusive currents have brought the membrane potential above threshold with a recovered fast inward inactivation gate, whereas the red dashed line indicates the fast inward current inactivation gate. For the induction of P2R in Figures 3(b) and 3(c), the three conditions of repolarization, recovery from inactivation, and excitation via diffusive currents are met. In Figure 3(a), P2R fails. Although recovered tissue is present, diffusive currents are not able to bring the recovered region above threshold of excitation.

The expanded views in Figure 3 also illustrate another important point: P2R does not originate exactly at the interface between the lost-dome and delayed-dome regions. Diffusive currents keep the membrane potential elevated in the lost-dome region close to the interface (with the precise extent determined by the diffusion coefficient), so that voltage-dependent recovery from fast inward current inactivation cannot be achieved. Thus, P2R of necessity must be initiated at a small distance from the interface, so that the membrane potential can recover initially to allow for recovery from inactivation and then subsequently be brought above the excitation threshold via diffusion from the delayed-dome region.

### 3.4. Generation of Phase-2 Reentry in Epicardial Tissue Sheets

Under similar conditions in two-dimensional epicardial layers, P2R can be produced and can lead to sustained reentry. In this case, normal epicardial cells were also included in addition to lost- and delayed-dome BrS epicardial regions. This is consistent with experimentally observed spatial gradients in protein expression and AP characteristics, which make the BrS phenotype more predominant in regions such as the region near the right ventricular outflow tract [24, 34].

**Figure 4:**
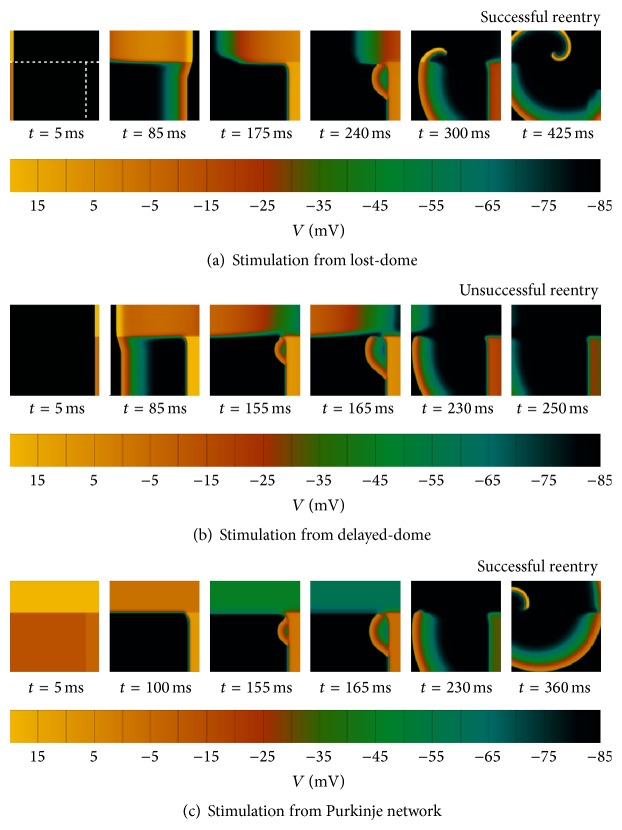
Induction of reentrant activity by phase-2 reentry (P2R) in epicardial tissue layers. The upper part of the 6 × 6 cm tissue represents normal epicardium, whereas the bottom part represents Brugada-affected epicardial tissue (left: lost-dome Brugada; right: delayed-dome Brugada). (a) Under stimulation from the lost-dome region, an antidromic P2R originates from the delayed-dome region, re-exciting the lost-dome epicardium and forming a stable reentry when the normal epicardial region recovers. (b) Under stimulation from the delayed-dome region, the orthodromic P2R is unable to find excitable normal epicardial tissue, and sustained reentry is not induced for this tissue size. (c) Under homogeneous epicardial activation, P2R develops analogously to the antidromic reentry case. Tissue coupling was decreased by 20% in order to accommodate the reentrant wave in the 6 × 6 cm domain. Color bar denotes transmembrane potential.

Figure 4 shows an example of a 6 × 6 cm domain with a fixed distribution of the three types of cells using Model 1, similar to experimental preparations of P2R in canine epicardium [9]. The influence of stimulation site in the successful generation of sustained reentry is demonstrated. In the case of antidromic P2R (Figure 4(a)), the initial wave propagates from the lost-dome to the delayed-dome region. The lost-dome region recovers quickly from this wave, whereas the normal epicardial region takes longer. After P2R generation, this dispersion of refractoriness facilitates formation of a stable reentry by delaying propagation of the P2R in the normal epicardial region. Once the normal region recovers, the wave curves, yielding a reentrant wave that continues to propagate back into the quickly recovering lost-dome region. In contrast, sustained reentry is not induced in the case of orthodromic P2R (Figure 4(b)), because, in a domain of this size, even when P2R occurs, it is unable to find excitable tissue into which the wavefront can turn and develop. If instead the entire epicardium is activated simultaneously (Figure 4(c)), simulating homogeneous activation from the Purkinje system, P2R remains inducible and proceeds to develop analogously to the antidromic reentry case.

### 3.5. Phase-2 Reentry in Reconstructed Ventricular Geometries

**Figure 5:**
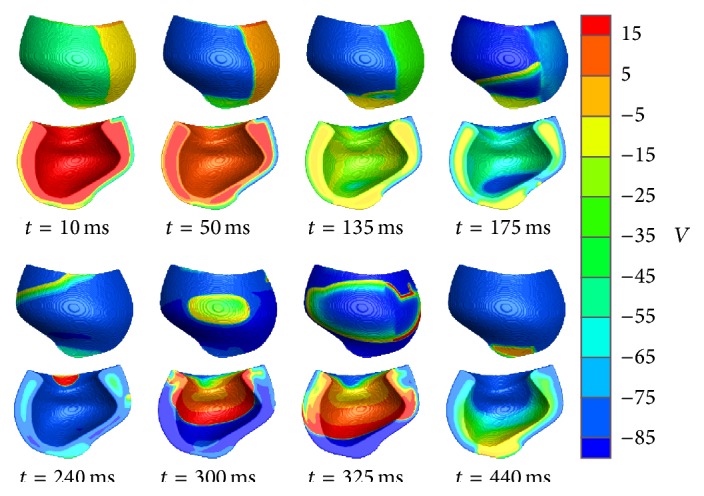
Induction of reentrant activity by phase-2 reentry (P2R) in the right ventricular free wall. Under endocardial stimulation, the Brugada epicardial delayed-dome region is able to initiate P2R in the lost-dome region (*t* = 135 ms). The P2R wavefront propagates from apex to base (*t* = 175 ms), where it reexcites the recovered endocardial layer (*t* = 240 ms). Afterwards, reentry develops transmurally (*t* = 300–440 ms). Epicardial and endocardial views of the right ventricular free wall are provided. Color bar denotes transmembrane potential. Epicardial regions are as follows: lost-dome Brugada, upper left; late-dome Brugada, lower left; and normal, right.

P2R can also initiate reentrant activity in reconstructed three-dimensional ventricular wall preparations, as shown in Figure 5. A distribution of normal and BrS epicardial regions, similar to that of the two-dimensional case, was used together with normal endocardial and midmyocardial cells and was incorporated into a model of the canine right ventricular free wall extracted from a full ventricular model [35]. Following endocardial stimulation of the ventricular wall, the entire epicardium is excited nearly simultaneously. However, the shorter APs in the lost-dome region recover quickly, and the delayed-dome region is able to initiate P2R, which propagates within the lost-dome region. The P2R wavefront is then able to reexcite the recovered endocardial tissue at the base of the ventricle, and reentry develops transmurally, newly activating the entire midmyocardial and epicardial ventricular layers.

## 4. Discussion

In this study, we unravel the key action potential and tissue coupling mechanisms underlying the induction of P2R in BrS. Beyond a marked dispersion of repolarization due to adjacent regions of lost-dome and delayed-dome Brugada-affected APs, our findings highlight that the successful achievement of P2R in BrS requires a specific balance of multiple additional factors. These include, at the level of the voltage profile of Brugada-affected APs, both the morphology of the delayed phase 2 of the AP and the relative duration of lost-dome and delayed-dome regions. Importantly, our results show for the first time the crucial role of cell-to-cell coupling strength in the triggering of P2R, through the modulation by diffusive currents of the recovery of tissue excitability. In addition, our investigations constitute the first simulation study of sustained reentry by P2R mechanisms in BrS, both in two-dimensional tissue layers and reconstructed three-dimensional ventricular wall preparations. These findings suggest the need of unaffected epicardial BrS regions for the successful establishment of reentrant circuits and that although both antidromic and orthodromic P2R can arise in one-dimensional cables, sustained reentrant activity is facilitated by the antidromic patterns in two-dimensional tissue layers. Finally, our results provide the first illustration of transmural reentry by P2R mechanisms in reconstructed ventricular geometries, where P2R is capable of reexciting a recovered basal endocardium and then developing through the rest of the ventricular wall.

Due to the increasing number of different mutations and ionic channels implicated in BrS, the evaluation of the morphological characteristics of Brugada-affected APs on the onset of P2R is achieved in the present contribution through the use of a minimal human AP model. Our model, in agreement with experimental recordings, allows among other characteristics for the alteration of the spike-and-dome configuration of the human epicardial AP, enabling the isolation of the role of the AP voltage profile without further assumptions on specific ionic currents. However, previous computational efforts have been directed at the understanding of BrS. The first simulation study at the ionic level using a biophysically detailed model was presented by Clancy and Rudy [29]. In their work, the authors showed that their simulated current reduction in mutant 1795insD sodium channels was able to produce a marked notch configuration of the AP in a single cell at fast pacing rates. Their mutant channel models were later extended by Bébarová et al. for the study of F2004L mutations in the SCN5A gene [15], showing discontinuous conduction in one-dimensional cables, but not P2R. In an approach different from that of Clancy and Rudy, the role of the L-type calcium current in BrS was studied by Miyoshi et al. [23], where a modified version of the Luo-Rudy model was also successfully used to replicate the features of BrS, including P2R in one-dimensional cables. Their approach was followed by the same group [36], as by Maoz et al. [37], to evaluate the propensity of P2R to arise from different sodium and transient potassium current density distributions. However, none of these studies addressed the investigation of sustained reentry by P2R mechanisms in either two- or three-dimensional preparations of BrS. Simulation of the Brugada ECG at the whole-heart level, again in the absence of P2R, was yet considered by Xia and coauthors [38].

In agreement with the aforementioned one-dimensional cable studies [23, 36, 37], our simulation results highlight the careful balance between the duration of the lost-dome and the voltage profile of the delayed-dome BrS AP regions for the successful initiation of P2R. Gradients in protein expression and AP characteristics have been extensively reported throughout the ventricles of many different species of mammals [11, 39, 40], including human [33, 41], that can be enhanced during cardiac alternans [42]. The existence of these gradients may be sufficient to obtain a particular distribution of AP morphologies in BrS in the right ventricular epicardial surface (delayed-dome and lost-dome regions), which is able to elicit reentrant activity in the form of P2R. As a result, the relative duration of APs between the lost-dome and delayed-dome regions is key for P2R generation, as the shorter the APs in the lost-dome region, the sooner the tissue can recover for a reexcitation. However, our results also show that a rapid delayed-dome activation (Model 1) is not an essential requirement for the onset of P2R but that APs of smoother dome activation (Model 2), as those experimentally recorded* in vivo* in the right ventricular outflow tract of BrS patients [24], can also successfully induce P2R when adjacent to BrS lost-dome regions.

Our findings indicate that P2R in BrS is mainly due to diffusive effects between epicardial regions of delayed-dome and lost-dome in the tissue. These results also indicate that two different stages have to be present in these diffusive effects. Initially, they must be relatively moderate in order to have little influence in the repolarization of the lost-dome region, allowing the tissue to recover excitability. However, immediately afterwards they must provide sufficient current injection in the lost-dome region of the tissue. Regardless of the AP upstroke amplitude, the only means to obtain such a distribution of diffusive effects in the interface between both regions is by the presence of a marked notch in the delayed-dome BrS AP. This notch must be not only deep, but also as wide as possible to yield the moderate effects of the first stage, and then followed by a delayed second upstroke as those in the APs of Figure 1, so that the delayed-dome region is able to bring the recovered region above the threshold of excitation.

Importantly, our work is the first simulation study analyzing the primal role of cell coupling in the triggering of P2R. Cell coupling is an important determinant of the voltage profile, which indicates whether it is possible to bring the membrane potential of a cell above the threshold of excitation by diffusive currents. Our simulation results illustrate that reduced electrical coupling facilitates initiation of P2R in BrS. Even though diffusive effects must be large in the second part of the reexcitation process, the most important stage of the mechanism is the recovery of tissue excitability. Diffusive effects between the lost-dome and delayed-dome regions initially must be as small as possible to promote this recovery, which is facilitated under conditions of decreased coupling, since larger portions of the lost-dome region can stay at their resting membrane potential without being pulled by the voltage profile of adjacent regions. Other geometrical [43, 44] and electrotonic [45] factors that are known to affect electrotonic coupling could play also a role.

Even though one-dimensional cables represent the simplest cellular arrangement for the theoretical investigation of P2R, diffusive mechanisms can be substantially different in the presence of transverse and transmural tissue coupling, where the injection of current from delayed-dome AP regions needs to be spread into an increasing bulk of surrounding myocytes. Moreover, the induction of a P2R wavefront does not necessarily guarantee the successful generation of sustained reentry, as highlighted in this contribution in the differences in reentry between antidromic/endocardial and orthodromic P2R activation patterns in epicardial layers (Figure 4).

In these simulated preparations, our results also show that P2R can only originate sustained reentrant activity when an appropriate distribution of AP morphologies is given in the tissue. Our configuration of AP morphologies (delayed-dome, lost-dome, and normal tissue) is similar to those investigated experimentally by Lukas and Antzelevitch in canine epicardium [9]. A delayed-dome configuration of the AP (in regions of the right ventricular outflow tract of approximately 4 cm in diameter) adjacent to areas of normal epicardial sites was first experimentally recorded* in vivo* in BrS patients by Kurita et al. [24]. Very recently, Zhang et al. [46] have also reported steep repolarization gradients in these areas through noninvasive ECG imaging in BrS patients compared to controls (105 ± 24 versus 7 ± 5 ms/cm). Our results, in agreement with previous modelling studies [23, 36, 37], show as well that even small variations in model parameters can easily produce the loss of dome in Brugada-affected APs. Circadian fluctuations in serum concentrations, together with spatially heterogeneous ionic and/or mutation expressions, might therefore yield transient distributions of AP morphologies like those hypothesized in this study for the successful generation of sustained reentry in this group of patients.

In addition, reduced coupling is also needed to justify the presence of the aforementioned large repolarization gradients reported in BrS patients [24, 46], which in principle would not be possible if electrical coupling was normal [47, 48]. These observations further support our findings on the role of a reduced electrical coupling as a precursor of P2R in BrS. Although traditionally BrS patients have been reported to have structurally normal hearts [1–3], significant structural modifications related to tissue uncoupling have been reported in some others, including interstitial fibrosis, right ventricular enlargement, myocyte disorganization, and abundant adipose tissue [19, 49–51], which are usually not detected using routine clinical diagnostic tests. Hence, although our analysis aimed to understand the mechanisms producing the successful initiation of P2R in BrS as a repolarization disorder (marked epicardial heterogeneity due to adjacent lost-dome and delayed-dome regions), our results also support the depolarization hypothesis in the form of a combined role of conduction and repolarization abnormalities in explaining the arrhythmogenesis of the disease [8, 20, 46].

From a modeling perspective, our work also illustrates the adaptation of a phenomenological human AP model for the study of genetic cardiomyopathies. Our model does not give independent descriptions for specific ion channels but rather focuses on the general properties of fast inward, slow inward, and slow-outward currents. Thus, it is difficult for this model to predict the role of specific currents. However, this conceptual framework is helpful in determining the physiological conditions necessary to produce Brugada-affected APs and P2R. It is possible for different ionic current alterations to combine to produce similar mesoscopic phenomena, and indeed our analysis of P2R is independent of specific mutations, which can be an advantage given that there may be multiple underlying genetic causes of BrS [6].

Finally, the role of structural abnormalities in the development of arrhythmias in BrS has yet to be unraveled. Both dispersion of repolarization and conduction delays could be maximized if local fibrosis or other mechanisms of myocyte uncoupling were present in tissue. Future work will investigate how ion channelopathies interact with structural derangements to promote arrhythmogenesis in the disease, for which novel modeling approaches aimed at incorporating into tissue simulations the macroscopic effects of structural heterogeneity [52], as well as descriptions of intersubject variability in both healthy and diseased conditions [53, 54], might greatly help in advancing our still limited understanding of the proarrhythmic mechanisms of the disease.

## Figures and Tables

**Table 1:** Parameter values used in the ionic model to produce Brugada syndrome action potentials. Values marked n/a are not applicable for the parameter set specified and can be set to any value.

Parameter	Normal Epi	Model 1	Model 2
*τ*_*v*1_^+^	1.4506	1.4506	3.33
*τ*_*v*1_^−^	60	60	60
*τ*_*v*2_^−^	1150	100	50
*τ*_*w*1_^+^	200	25	25
*τ*_*w*2_^+^	200	125	230
*τ*_*w*1_^−^	60	60	595
*τ*_*w*2_^−^	15	15	25
*τ*_*s*1_	2.7342	2.7342	2.7342
*τ*_*s*2_	16	35	25.212
*τ*_fi_	0.11	0.04	0.05
*τ*_*o*1_	400	400	8.8543
*τ*_*o*2_	6	6	8.8543
*τ*_so1_	30.0181	30.0181	193.9115
*τ*_so2_	0.9957	0.9957	0.564
*τ*_si1_	1.8875	7.5476	5.8343
*τ*_si2_	1.8875	1.8875	0.1567
*τ*_*v∞*_	0.006	0.13	0.12
*θ*_*v*_	0.3	0.3	0.13
*θ*_*v*_^−^	0.006	0.006	0.006
*θ*_*v∞*_	0.006	2	2
*θ*_*w*_	0.13	0.13	0.13
*θ*_*w∞*_	0.13	0.13	0.12
*θ*_so_	0.13	0.13	0.2564
*θ*_si_	0.13	0.13	0.13
*θ*_*o*_	0.006	0.006	0.006
*θ*_*s*_	0.13	0.13	0.36
*k*_*w*_^+^	n/a	5.7	6.2
*k*_*w*_^−^	65	65	65
*k*_*s*_	2.0994	5.8	4.825
*k*_so_	2.0458	2.0458	3.1353
*k*_si_	n/a	97.8	4.7317
*u*_*w*_^−^	0.03	0.03	0.03
*u*_*s*_	0.9087	0.35	0.4363
*u*_*o*_	0	0	0
*u*_*u*_	1.55	1.0	0.6
*u*_so_	0.65	0.65	0.11
*s*_*c*_	n/a	0.7175	0.642
*w*_*c*_^+^	n/a	0.15	0.464
*w*_*∞*_^*∗*^	0.94	0.94	0.94
